# Dynamics of HBsAb persistence and associated BCR IgG-H CDR3 repertoire features in ultra-high versus extremely low responders to HBV vaccination

**DOI:** 10.3389/fimmu.2025.1587553

**Published:** 2025-07-04

**Authors:** Ting-Wen Yang, Li-Na Sun, Ting Zhang, Yu-Rong Pan, Jun Li, Yang-Yang Li, Zhi-Yong Han, Wan-Bang Sun, Xin-Sheng Yao, Xiao-Yan He

**Affiliations:** ^1^ Department of Immunology, Center of Immunomolecular Engineering, Innovation & Practice Base for Graduate Students Education, Zunyi Medical University, Zunyi, China; ^2^ Department of Laboratory Medicine, The Second People's Hospital of Guiyang City, Guiyang, China

**Keywords:** BCR-H CDR3 repertoire, hepatitis B virus, high-throughput sequencing, HBV vaccine, HBsAb

## Abstract

**Objective:**

This study aimed to investigate the duration of hepatitis B surface antibody (HBsAb) level maintenance and characterize the corresponding IgG-H CDR3 repertoire in volunteers exhibiting either ultra-high or extremely low HBsAb levels following hepatitis B virus (HBV) vaccination.

**Methods:**

This study was designed to monitor the longitudinal changes in HBsAb levels at multiple time points post-vaccination in healthy volunteers exhibiting either ultra-high or extremely low antibody responses following HBV vaccination. Furthermore, we employed high-throughput sequencing technology to conduct a comprehensive analysis of the compositional differences in the IgG-H CDR3 repertoire. Peripheral blood samples were collected at four distinct phases: pre-vaccination, post-second vaccination, post-third vaccination, and during the follow-up period, to characterize the dynamic changes in the antibody repertoire.

**Results:**

The longitudinal analysis revealed distinct patterns in antibody response kinetics between the two groups. In the ultra-high responder cohort, a peak HBsAb level (25,354 ± 17,993 mIU/mL) was observed following the second vaccine dose. This was followed by a gradual decline, with mean levels stabilizing at 11,356 ± 9,098 mIU/mL post-third dose and 4,229 ± 2,694 mIU/mL at the 4-year follow-up. The ultra-high hepatitis B surface antibody level group (Group H) exhibited a decrease in IgG-H CDR3 diversity after the second vaccination, followed by an increase after the third vaccination. Compared to the extremely low hepatitis B surface antibody level group (Group L), Group H showed higher characteristic immunoglobulin heavy chain variable region V gene (*IGHV*) usage frequencies in the CDR3 repertoire after both vaccinations than before vaccination. Additionally, the average mutation rate of the IgG-H CDR3 repertoire in Group H was slightly higher than that in Group L after the third vaccination. Notably, multiple samples from Group H revealed common conserved CDR3 region motifs associated with HBV, as reported in the literature: “YGLDV”, “DAFD”, “YGSGS”, “GAFDI”, and “NWFDP”.

**Conclusion:**

The prolonged maintenance of ultra-high antibody levels induced by recombinant HBV vaccines in individuals may be closely related to the characteristic *IGHV* usage and mutation frequency in individual responses. These findings could provide novel insights into the complex mechanisms of HBV vaccines and HBV infection.

## Introduction

Hepatitis B virus (HBV) infection is primarily prevented through immunization with recombinant HBV vaccines. According to the World Health Organization (WHO) criteria, an HBsAb level ≥10 mIU/mL is considered indicative of protective immunity following HBV vaccination (1). While over 90% of vaccine recipients who complete the full vaccination schedule develop protective levels of hepatitis B surface antibodies (HBsAb) that may persist for decades or throughout their lifetime, approximately 5-10% of individuals exhibit vaccine hyporesponsiveness, failing to mount adequate antibody responses (2). The immunological mechanisms underlying this interindividual variability in HBsAb production and the corresponding B-cell response heterogeneity remain poorly understood. Comprehensive sequencing and analysis of the B-cell receptor (BCR) CDR3 repertoire in vaccine recipients with divergent antibody responses may provide novel insights into the molecular basis of HBV vaccine responsiveness and offer a unique perspective on the relationship between BCR repertoire characteristics and vaccine-induced immunity.

Vaccination against the HBV is the most crucial measure for preventing HBV infection. Both domestic and international statistics and studies indicate that only about 90% of the population can produce HBsAb after HBV vaccination. Among them, 10% to 20% of healthy adults produce low levels of protective antibodies (anti-HBs), showing a low-response state (3), and they remain susceptible to HBV infection. Approximately 10% of healthy individuals do not produce HBsAb at all, exhibiting a non-response state (4). The different response states of individuals are closely related to B cells. In some individuals, HBsAb levels can reach up to 100,000 IU/L after HBV vaccination (5), providing strong protection. However, the mechanisms underlying the individual differences in HBV responses have not yet been fully elucidated (6). Doedée et al. (7) compared the differences in HBsAg-specific memory B cells and IgG levels between individuals with HBsAb titers > 20,000 IU/L (hyper-response) and those with HBsAb titers < 1,500 IU/L (high-response) after HBV vaccination. They found that before and after the second vaccination, the number of HBsAg-specific memory B cells in hyper-responders was significantly higher than that in high-responders, and plasma IgG levels were significantly correlated with the number of HBsAg-specific memory B cells. This suggests that the difference in anti-HBs titers between hyper-responders and high-responders is associated with the number of memory B cells. Van Damme et al. (8) demonstrated that after HBV vaccination, B cell-mediated immune memory can provide long-term protection even after the induced HBsAb levels decline (9). This long-term protection can be assessed by detecting the *in vitro* activation of HBsAg-specific B cells.

The current understanding of the relationship between HBV vaccination and BCR-H CDR3 repertoire dynamics remains limited. Following immunization, a marked interindividual variability in immune responses is observed, with some recipients developing ultra-high HBsAb titers (>10,000 mIU/mL) while others exhibit hyporesponsiveness (HBsAb <10 mIU/mL) (7). Emerging evidence indicates significant alterations in BCR gene composition, including preferential *V(D)J* gene segment usage, distinct gene family expression patterns, and repertoire diversity changes following vaccination (10–14). However, a systematic comparison of BCR repertoire characteristics between these two phenotypic extremes - particularly in ultra-high responders where research remains scarce - has yet to be comprehensively investigated. This knowledge gap hinders our understanding of the molecular mechanisms underlying differential vaccine responsiveness.

Building upon our previous investigations into HBV vaccine-induced BCR CDR3 repertoire dynamics, we have identified a potential correlation between high-frequency CDR3 generation patterns and IgG affinity maturation processes during B-cell responses. Notably, in a subset of vaccine recipients exhibiting exceptional seroconversion (HBsAb >10,000 mIU/mL) - a phenomenon whose underlying mechanisms remain elusive - we observed consistent preferential usage of specific *IGHV*, immunoglobulin heavy chain variable region D gene (*IGHD*), and immunoglobulin heavy chain variable region J gene (*IGHJ*) combinations in their BCR repertoires (15, 16). Based on these preliminary findings, the current study was designed to longitudinally monitor healthy vaccine recipients with polarized antibody responses (ultra-high versus extremely low HBsAb levels) and perform a comprehensive comparative analysis of their antibody persistence profiles following HBV vaccination. Peripheral blood samples were systematically collected at predetermined time points for comprehensive analysis of IgG-H CDR3 repertoire composition and diversity. This study was designed to elucidate the cellular and molecular mechanisms underlying the generation of polarized HBsAb responses (ultra-high versus extremely low) following HBV vaccination. Through this investigation, we aim to establish robust comparative baseline data and provide novel mechanistic insights into HBV vaccine immunology, with particular emphasis on exceptional responders exhibiting vaccine-induced hyper-seroconversion. Furthermore, our findings may contribute to the identification of potential therapeutic targets and inform the development of innovative strategies for generating high-affinity, neutralizing antibodies through BCR repertoire engineering.

## Materials and methods

### Sample collection and vaccination

This study employed a rigorous screening process to enroll eligible participants from the Zhuhai Campus of Zunyi Medical University. Among 222 initially screened students, 49 seronegative volunteers (testing negative for all five HBV markers: HBsAg, HBsAb, HBeAg, HBeAb, and HBcAb) without history of infectious, genetic, or autoimmune diseases were recruited. Participants received the standard three-dose recombinant HBV vaccination regimen (0, 1, and 6 months) at the Sanzao Township Health Center, Jinwan District, Zhuhai City, Guangdong Province. Each intramuscular dose consisted of 20μg recombinant hepatitis B vaccine, with the first two doses administered using GlaxoSmithKline (USA) products and the third dose from Dalian Hissen Bio-Pharm Co., Ltd. (China), ensuring consistent antigenic content across vaccinations.

Based on quantitative HBsAb measurements following the second vaccination, we stratified participants into two distinct cohorts: Group H (HBsAb >10,000 mIU/mL) and Group L (HBsAb <10 mIU/mL), with five volunteers randomly selected from each group for detailed analysis. All volunteers were healthy young students who had never received HBV vaccination before and had no history of infection, autoimmune diseases, genetic disorders, obesity, or metabolic syndrome. Longitudinal peripheral blood samples were collected at four predetermined time points: baseline (T1: pre-vaccination), 2 weeks after the second dose (T2), 1 month after the third dose (T3), and during long-term follow-up (T4: 4 years post-vaccination completion). The study protocol was conducted in full compliance with ethical standards, having obtained written informed consent from all participants and approval from the Institutional Review Board of Zunyi Medical University (Ethical Approval Number: (2020) 1-009).

### Detection of HBsAb levels

Serological screening was performed using enzyme-linked immunosorbent assay (ELISA) (**Supplementary Table S1**) (Beijing Wantai Biological Pharmacy Enterprise Co., Ltd. HBsAg Detection Kit (National Medical Device Registration No. 20153400391): Sensitivity: 99.2% (95% CI: 98.5–99.6%); Specificity: 99.5% (99.0–99.8%). HBsAb Detection Kit (National Medical Device Registration No. 20153400392): Sensitivity: 98.8% (97.9–99.3%), Specificity: Typically extremely high, approaching or exceeding 99%; Detection range: 2–1000 mIU/mL. HBeAg Detection Kit (National Medical Device Registration No. 20153400394): Sensitivity: 98.5% (97.6–99.1%); Specificity: 99.0% (98.4–99.4%). HBeAb Detection Kit (National Medical Device Registration No. 20153400395): Sensitivity: 98.3% (97.3–98.9%), Specificity: Typically extremely high, approaching or exceeding 99%. HBcAb Detection Kit (National Medical Device Registration No. 20153400396): Sensitivity: 98.7% (97.8–99.2%); Specificity: 99.1% (98.6–99.4%)) to quantify all five hepatitis B markers (HBsAg, HBsAb, HBeAg, HBeAb, and HBcAb) prior to vaccination initiation. For immunological analysis, 3 mL serum samples were collected from all 10 enrolled volunteers at two critical time points: post-second vaccination and post-third vaccination. During the 4-year follow-up period, 2 mL plasma samples were obtained from 9 available participants. Quantitative measurement of HBsAb levels was conducted using chemiluminescent microparticle immunoassay (CMIA) (Sensitivity: ≥ 99%; specificity: ≥ 99.5%), with all assays performed in duplicate to ensure measurement reliability.

### Principles and procedures for constructing the BCR-H CDR3 repertoire

Peripheral blood mononuclear cells (PBMCs) were isolated from collected samples using density gradient centrifugation, followed by total RNA extraction with TRIzol reagent. The extracted RNA was subsequently reverse transcribed into complementary DNA (cDNA) using a high-efficiency reverse transcription kit. For BCR repertoire analysis, we employed a standardized protocol for human BCR-H CDR3 repertoire preparation, wherein target regions were amplified through nested PCR with gene-specific primers. The amplified products were then subjected to high-throughput sequencing (HTS) using the Illumina HiSeq platform (2 × 150 bp paired-end reads), with all sequencing services provided by BGI (Shenzhen, China) (17–19).

### Data filtering and sorting

The high-throughput sequencing data of BCR-H CDR3 repertoires were processed and analyzed using a comprehensive bioinformatics pipeline. Initial quality control and repertoire characterization were performed using ImmuHub and IMGT’s High-V-QUEST tools, with subsequent sequence clustering based on stringent criteria: (1) identical *IGHV* and *IGHJ* gene segment usage, (2) matching CDR3 amino acid sequence lengths, and (3) allowance of ≤1 amino acid mismatch per 12 residues (20). The clustered repertoire data were then subjected to multidimensional feature analysis, including: (i) CDR3 repertoire diversity indices, (ii) *IGHV-IGHJ* gene segment usage patterns and pairing preferences, (iii) somatic hypermutation profiles, and (iv) repertoire overlap characteristics. The raw sequencing data have been deposited in the NCBI Sequence Read Archive under accession number PRJNA1226991 (https://www.ncbi.nlm.nih.gov/bioproject/PRJNA1226991/). Additional processed data, including CDR3 clustering analysis results, are available upon reasonable request from the corresponding author.

### Statistical analysis and tools

Statistical analyses were conducted using IBM SPSS Statistics 22 (IBM Corp., Armonk, NY, USA). Non-parametric tests were employed based on data distribution characteristics: the Mann-Whitney U test for comparisons between two independent groups and the Kruskal-Wallis test for multiple group comparisons. All statistical tests were performed as two-tailed analyses with a predetermined significance threshold of α = 0.05. The following notation was used to indicate statistical significance levels: *P < 0.05, **P < 0.01, and ***P < 0.001. Effect sizes were calculated for significant findings to assess the magnitude of observed differences.

## Results

### Levels of hepatitis B serological markers in HBV vaccinated volunteers

Following the second dose, HBsAb results were obtained from 45 participants, as 4 were lost to follow-up. Of these, 5 (11.1%) exhibited HBsAb levels below 10 mIU/mL (**Supplementary Table S2**). At T1 (pre-vaccination), T2 (post-second dose), and T3 (post-third dose), five hepatitis B serological markers (HBsAg, HBsAb, HBeAg, HBeAb, and HBcAb) were longitudinally monitored in 10 volunteers, while at T4 (4-year follow-up), monitoring was conducted in 9 volunteers (**Table 1**). Baseline screening using enzyme-linked immunosorbent assay (ELISA) confirmed seronegativity for all five markers in all participants. Group H demonstrated robust seroconversion, with peak HBsAb levels reaching 25,354 ± 17,993 mIU/mL at T2. These levels subsequently stabilized at 11,356 ± 9,098 mIU/mL (T3) and 4,229 ± 2,694 mIU/mL (T4), indicating sustained antibody persistence. In contrast, in Group L, all 5 volunteers had HBsAb levels below the protective threshold of 10 mIU/mL after the second dose. Following the third dose, 3 out of these 5 volunteers showed increased HBsAb levels above 10 mIU/mL, though still at low concentrations. Four years after completing the full HBV vaccination series, only 1 volunteer in Group L maintained HBsAb levels above 10 mIU/mL.

**Table 1:** Levels of five markers of hepatitis B in volunteers.

Sample	Sex	Age	T1^a^					T2^a^	T3^a^	T4^a^
			HBsAg	HBsAb	HBeAg	HBeAb	HBcAb	HBsAb (mIU/mL)^b^	HBsAb (mIU/mL)^b^	HBsAb (mIU/mL)^b^
H1	Male	20	(-)	(-)	(-)	(-)	(-)	61253.73	28825.22	7615.33
H2	Female	22	(-)	(-)	(-)	(-)	(-)	17896.48	10739.97	4418
H3	Female	21	(-)	(-)	(-)	(-)	(-)	16920.24	6909.83	2237.95
H4	Male	20	(-)	(-)	(-)	(-)	(-)	16569.18	7556.21	6565.24
H5	Female	19	(-)	(-)	(-)	(-)	(-)	14130.51	2747.09	309.01
L1	Female	20	(-)	(-)	(-)	(-)	(-)	5.82	88.72	7.51
L2	Male	20	(-)	(-)	(-)	(-)	(-)	5.06	435.6	51.65
L3	Male	20	(-)	(-)	(-)	(-)	(-)	2.78	4.62	0.17
L4	Female	19	(-)	(-)	(-)	(-)	(-)	1.15	4.55	0.16
L5	Male	24	(-)	(-)	(-)	(-)	(-)	0.93	10.97	Withdrawal

(a) T1 is the first blood collection i.e. before vaccination, the five hepatitis B items were detected by ELISA, (-) indicates negative; T2 is the second blood collection i.e. 2 months after the second vaccination; T3 is the third blood collection i.e. 6 months after the third vaccination; T4 is the fourth blood collection i.e. four years after the full vaccination. The volunteers were numbered together with the group and the time point of blood collection in the follow-up analysis, e.g., sample 1 before vaccination in the ultra-high HBsAb level group was numbered T1-H1. (b) HBsAb: Hepatitis B surface antibody, reference range (0-9.99) mIU/mL.

### IgG-H CDR3 repertoire sequence count

Comprehensive BCR-H CDR3 repertoire profiling was performed on all samples through high-throughput sequencing (HTS), generating an average of 2,892,473 raw sequences per sample. Following quality control and preprocessing, sequence alignment and clustering were performed using established bioinformatics pipelines (12, 13, 21). Subsequent analysis using ImmuHub and IMGT’s High-V-QUEST tools revealed an average of 614,337 (range: 11,084-2,317,381) high-quality IgG-H CDR3 sequences per sample, corresponding to 68,021 (range: 4,260-823,216) unique clonotypes. Following rigorous clustering criteria (identity thresholds: *IGHV/IGHJ* gene usage, CDR3 length, and ≤1 amino acid mismatch per 12 residues), an average of 39,215 (range: 3,651-94,544) analyzable clusters were obtained per sample for the IgG-H CDR3 repertoire (**Supplementary Table S3**). These processed clusters formed the basis for subsequent repertoire diversity and clonality analyses.

### The diversity and clonal expansion of the IgG-H CDR3 repertoire

In high-throughput sequencing (HTS) analyses of immune repertoire diversity, the inverse Simpson’s diversity index (1/Ds) has emerged as a robust and widely adopted metric for quantifying BCR/TCR CDR3 repertoire heterogeneity across various physiological and pathological conditions (22–24). This index provides a quantitative measure of repertoire diversity, where higher 1/Ds values (approaching the total number of unique sequences) indicate increased diversity with limited clonal expansion, while lower values suggest repertoire contraction with dominant clonal amplification. The index is calculated using the following equation: 1/Ds = 1/∑{ni(ni-1)}/{N(N-1)}, where ni represents the frequency of the ith unique sequence and N denotes the total number of sequences in the repertoire. This metric has been particularly valuable for comparative analyses of immune repertoire dynamics in response to vaccination, infection, and immunotherapeutic interventions.

**Figure 1:**
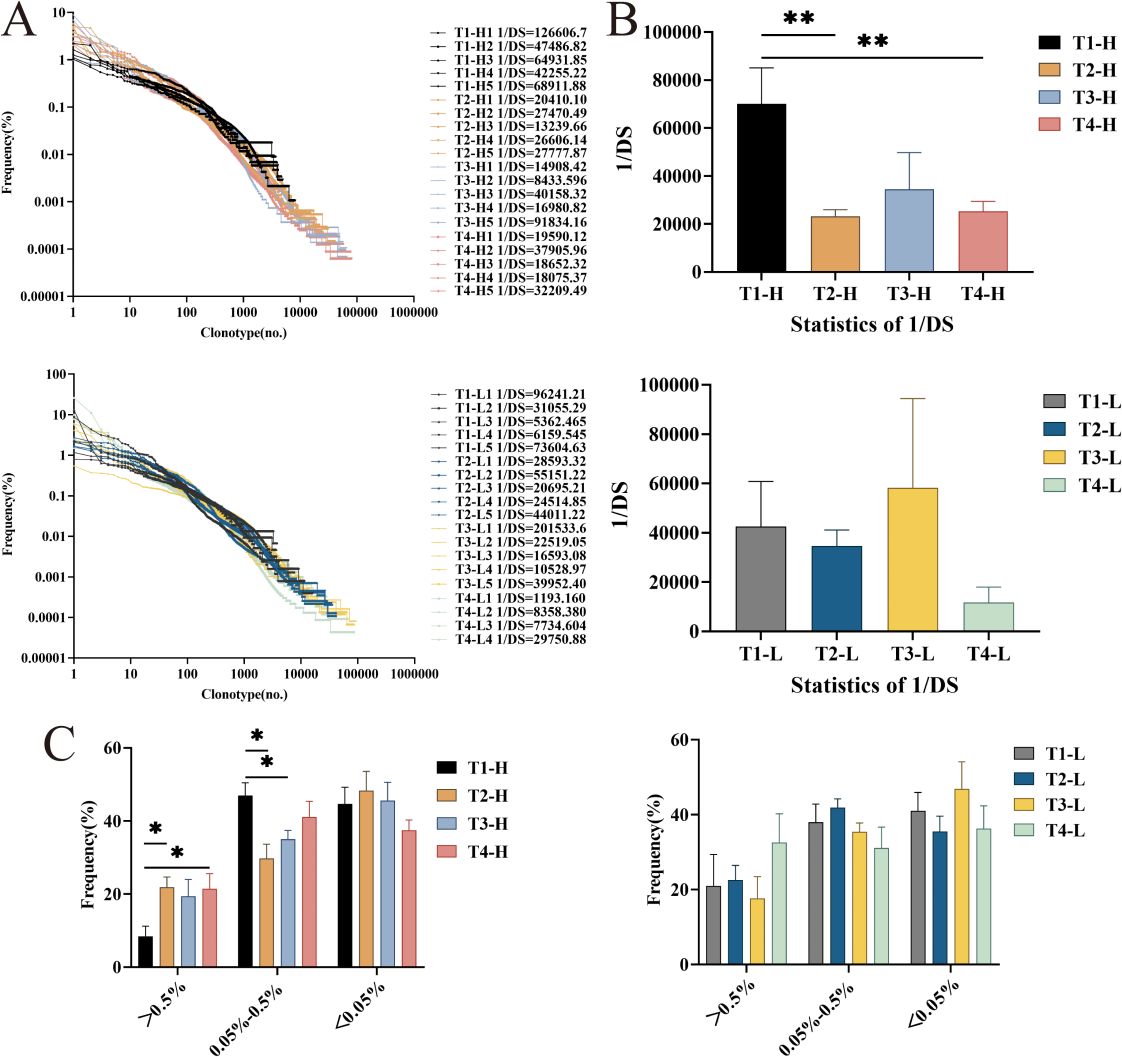
The diversity and the distribution of clones with different proportions of IgG-H CDR3 repertories in group H and group L. **(A)** Overall clonal distribution; **(B)** Statistical analysis of 1/DS; **(C)** Statistical analysis of the different proportions of clone distribution. Note. In the figure **(A, B)**, the top part shows results for group H and the bottom part for group L. In the **(C)**, the left part shows results for group H and the right part for group L. (Kruskal-Wallis test, *P < 0.05, **P < 0.01, ***P < 0.001).

Comparative analysis of clonal expansion patterns revealed no significant differences between Group H and Group L across all time points: pre-vaccination (T1), post-second vaccination (T2), post-third vaccination (T3), and 4-year follow-up (T4). However, longitudinal assessment of repertoire diversity using the 1/Ds demonstrated distinct temporal dynamics between the groups. In Group H, we observed a significant reduction in 1/Ds following the second vaccination (P = 0.009 vs. baseline), which subsequently rebounded after the third vaccination. Notably, the 4-year follow-up measurements in Group H remained significantly different from baseline levels (P = 0.009). While Group L exhibited similar temporal trends in 1/Ds values (decrease at T2 followed by increase at T3), these changes did not reach statistical significance (**Figures 1A, B**). These findings suggest that while both groups share similar clonal expansion patterns, they exhibit differential repertoire diversity dynamics in response to HBV vaccination.

Clusters with a clonal frequency ≥ 0.5% were defined as highly expanded clones (HEC) clusters, those with a clonal frequency between 0.05% and 0.5% were defined as intermediate expanded clones (MEC) clusters, and those with a clonal frequency ≤ 0.05% were defined as low expanded clones (LEC) clusters (25–27). The study found that the HEC clusters in the group H increased after the second vaccination, the third vaccination, and four years post-vaccination. Statistically significant differences were observed in the HEC clusters between pre-vaccination and post-second vaccination, as well as between pre-vaccination and four years post-vaccination (P = 0.028, P = 0.047). For the MEC clusters, statistically significant differences were noted between pre-vaccination and post-second vaccination, as well as between pre-vaccination and post-third vaccination (P = 0.028, P = 0.028). In the group L, the HEC clusters showed little change after the second and third vaccinations but increased four years post-vaccination, though this increase did not reach statistical significance (**Figure 1C**).

### Characteristic usage and pairing of *IGHV* and *IGHJ* genes in the IgG-H CDR3 repertoire

During the ontogeny of B lymphocytes, each gene segment exists in multiple distinct copies, and the process of gene rearrangement facilitates diverse combinations of these segments. This phenomenon of combinatorial diversity serves as a fundamental mechanism that significantly contributes to the heterogeneity of the BCR-H CDR3 repertoire. The analytical findings pertaining to the characteristic *IGHV* and *IGHJ* genes within the IgG-H CDR3 repertoires of both group H and group L are delineated as follows.

**Figure 2:**
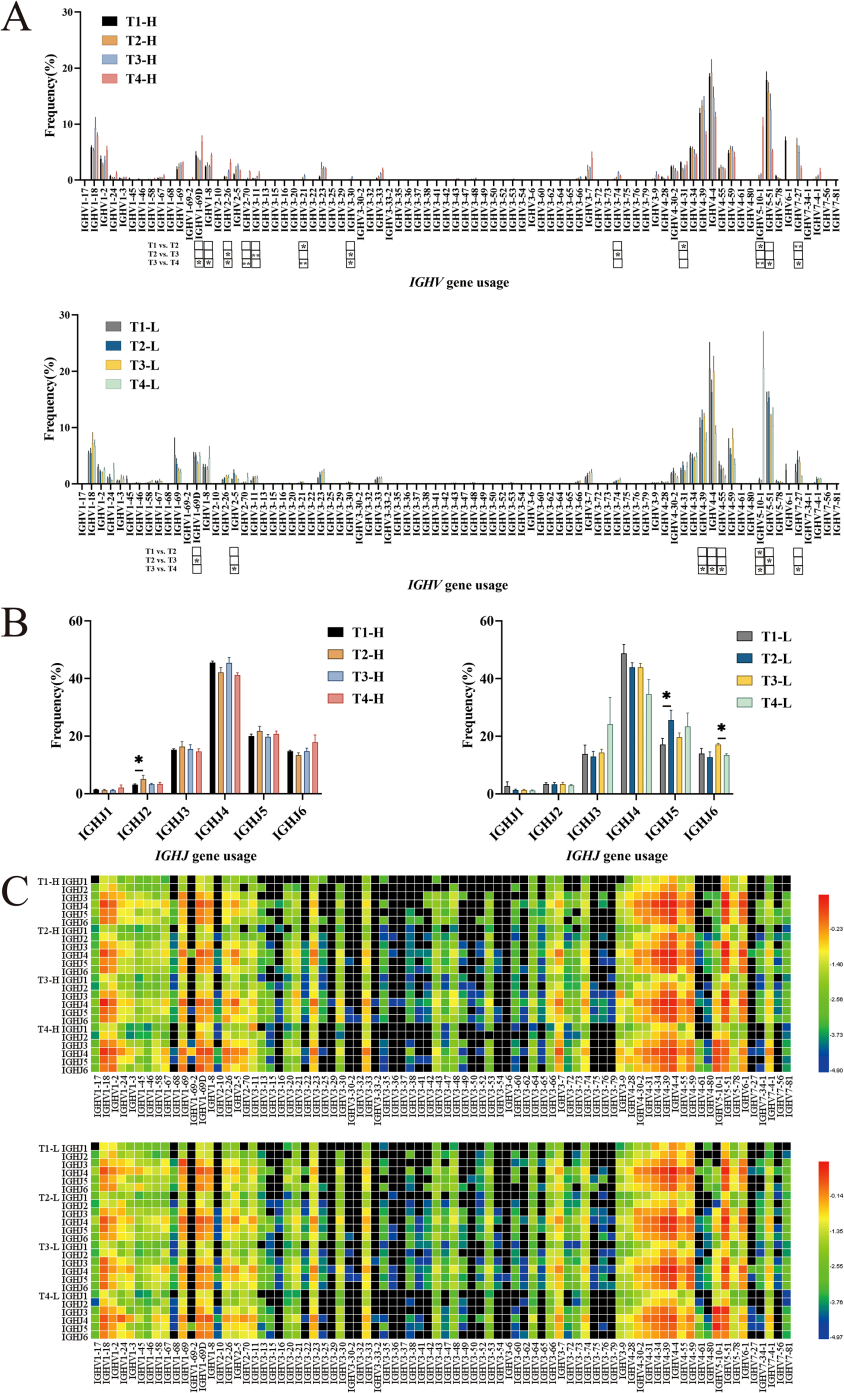
Analysis of *IGHV*, *IGHJ* usage and *IGHV*-*IGHJ* pairing in group H and group L. **(A)** *IGHV* usage. **(B)** *IGHJ* usage. **(C)** Pairing of *IGHV*-*IGHJ* gene. Note. In the figure **(A, C)**, the top part shows results for group H and the bottom part for group L. In the figure **(B)**, the left part shows results for group H and the right part for group L. (Kruskal-Wallis test, *P < 0.05, **P < 0.01, ***P < 0.001).

*IGHV* gene frequencies: In the group H, the usage frequencies of *IGHV1-18*, *IGHV2-26*, *IGHV2-5*, *IGHV3-21*, *IGHV3-23*, *IGHV3-33*, *IGHV3-7*, *IGHV3-74*, *IGHV3-9*, and *IGHV7–27* were higher post-vaccination compared to pre-vaccination. In contrast, the group L showed little difference in the usage frequencies of these genes before and after vaccination. Statistical analysis revealed differences in the usage of *IGHV3-21, IGHV4-31*, *IGHV5-10-1*, and *IGHV7–27* between pre-vaccination and post-second vaccination in the group H. Differences were observed in the usage of *IGHV2-26*, *IGHV3-11*, *IGHV3-30*, and *IGHV3–74* between the second and third vaccinations. Four years post-vaccination, the usage of *IGHV1-69D*, *IGHV1-8*, *IGHV2-26*, *IGHV2-70*, and *IGHV5-10–1* significantly increased, while the usage of *IGHV3-21*, *IGHV3-30*, *IGHV5-51*, and *IGHV7–27* significantly decreased. In the group L, differences were observed in the usage of *IGHV5-10–1* between pre-vaccination and post-second vaccination; differences were also noted in the usage of *IGHV1-69D* and *IGHV5–51* between the second and third vaccinations. Four years post-vaccination, only the usage of *IGHV5-10–1* significantly increased, while the usage of *IGHV2-5*, *IGHV4-39*, *IGHV4-4*, *IGHV4-55*, and *IGHV7–27* significantly decreased (**Figure 2A**).

*IGHJ* gene frequencies: The usage of *IGHJ* genes in the group H and the group L showed little difference, with the order being *IGHJ4* > *IGHJ5* > *IGHJ6* > *IGHJ3* > *IGHJ2* > *IGHJ1*. Statistical analysis revealed a difference in the usage of *IGHJ2* between pre-vaccination and post-second vaccination in the group H. In the group L, a difference was observed in the usage of *IGHJ5* between pre-vaccination and post-second vaccination, and the usage of *IGHJ6* significantly decreased four years post-vaccination (**Figure 2B**).

*IGHV-IGHJ* gene pairing: The heatmap analysis revealed that the *IGHV1-18-IGHJ4, IGHV4-4-IGHJ4, and IGHV6-1-IGHJ4* pairings exhibited slightly higher frequencies in Group H compared to Group L (**Figure 2C**).

### IgG-H CDR3 repertoire CDR3 length distribution and amino acid usage

**Figure 3:**
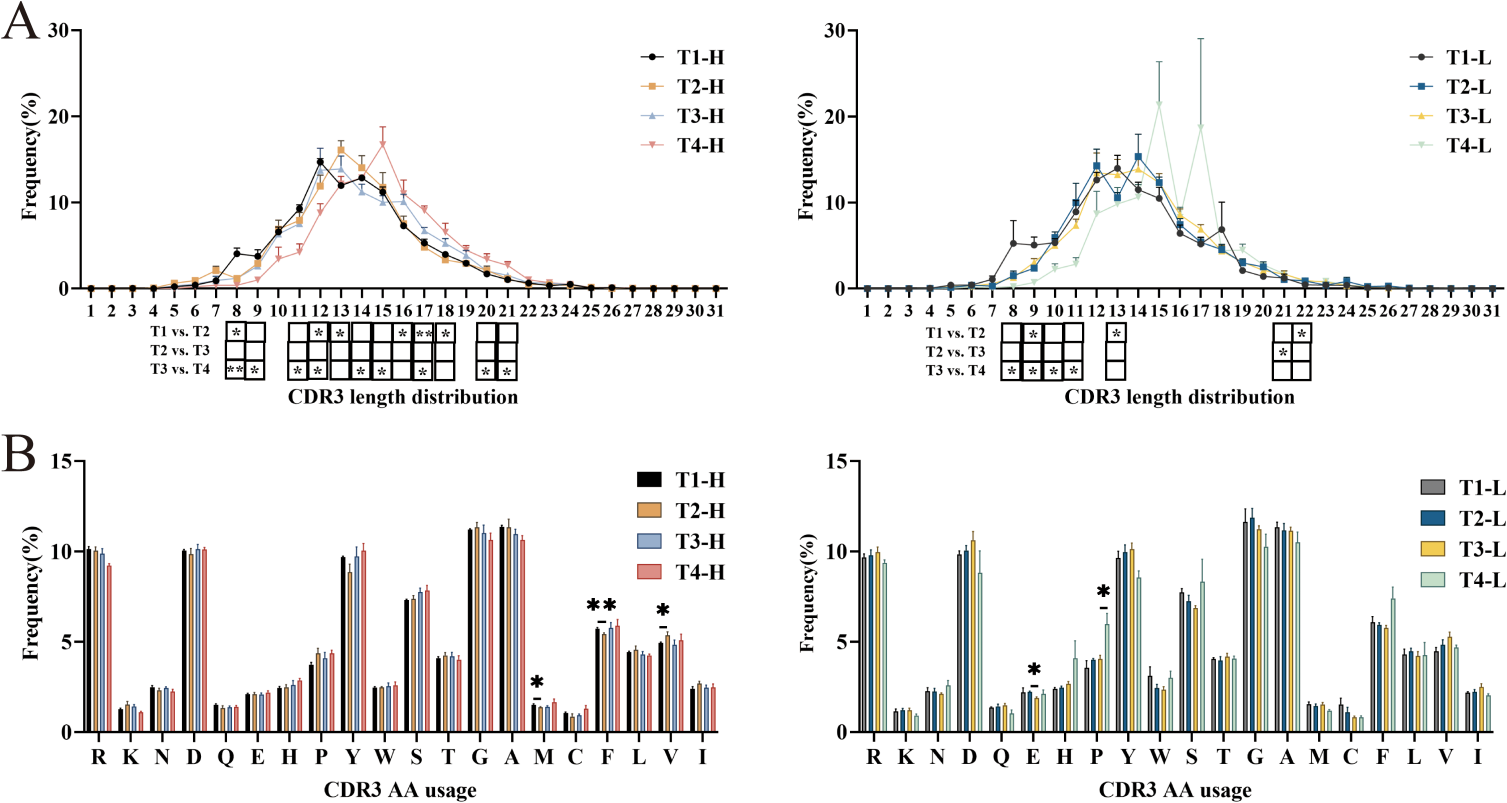
Analysis of CDR3 length distribution and Amino acids usage of IgG-H CDR3 repertoire in group H and group L. **(A)** CDR3 length distribution of IgG-H CDR3. **(B)** Amino acids usage of IgG-H CDR3. Note. In the figure **(A, B)**, the left part shows results for group H and the right part for group L. (Kruskal-Wallis test, *P < 0.05, **P < 0.01, ***P < 0.001).

The diversity of the CDR3 repertoire is substantially enhanced by structural variations in the CDR3 region, which arise from differences in both length and amino acid composition. Quantitative analysis of CDR3 length distribution patterns demonstrated an approximately normal distribution in both experimental groups. Notably, the length profiles remained consistent across three critical time points: pre-vaccination, post-second vaccination, and post-third vaccination, with the majority of sequences (12–15 amino acids in length) maintaining stable proportions. However, longitudinal analysis revealed a remarkable shift in the repertoire composition four years post-vaccination, with both group H and group L exhibiting significant clonal expansion of longer CDR3 sequences, predominantly ranging from 14 to 16 amino acids in length (**Figure 3A**).

Analysis of amino acid usage in both groups revealed that both groups frequently utilized arginine (R), aspartic acid (D), tyrosine (Y), serine (S), glycine (G), and alanine (A). Among these, except for glycine (G) and alanine (A), the rest are hydrophilic amino acids. Statistical analysis of each group separately showed that in the group H, there were statistically significant differences in the usage of methionine (M) (P = 0.047), phenylalanine (F) (P = 0.009), and valine (V) (P = 0.047) between pre-vaccination and post-second vaccination. In the group L, there was a statistically significant difference in the usage of glutamic acid (E) (P = 0.016) between post-second vaccination and post-third vaccination, and a statistically significant difference in the usage of proline (P) (P = 0.014) between post-third vaccination and four years post-vaccination. No significant differences were observed in the usage of other amino acids (**Figure 3B**).

### Somatic hypermutation, insertions, and deletions in high-frequency clonal sequences of the IgG-H CDR3 repertoire

**Figure 4:**
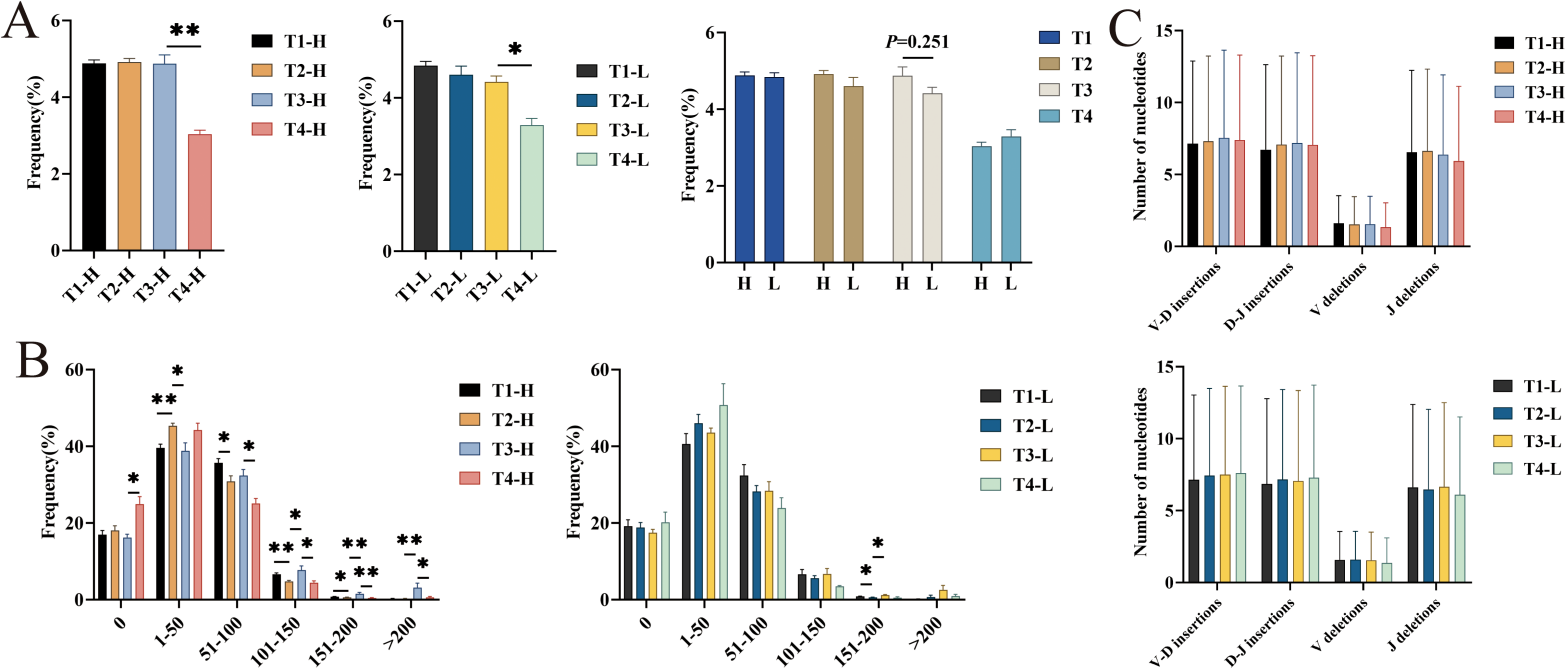
Analysis of average mutation rate, different numbers of high mutation nucleotide insertions and deletions in IgG-H CDR3 repertoire. **(A)** Average mutation rate in group H and group L; **(B)** Different numbers of high mutation (per 1000 amino acids) in group H and group L; **(C)** The average number of nucleotide insertions and deletions of sample 1 in group H and group L. Note. In the figure **(A, B)**, the left part shows results for group H and the right part for group L. In the figure **(C)**, the top part shows results for group H and the bottom part for group L. (Kruskal-Wallis test, *P < 0.05, **P < 0.01, ***P < 0.001).

Antigenic stimulation triggers somatic hypermutation (SHM), a process characterized by the accumulation of point mutations in the variable (V) region genes of B cells. This molecular mechanism significantly contributes to the diversification of the CDR3 repertoire through the introduction of additional sequence variations. Longitudinal analysis of mutation rates revealed comparable patterns in both cohorts across three critical time points: pre-vaccination, post-second vaccination, and post-third vaccination. However, a notable decline in mutation frequency was observed at the four-year post-vaccination time point. Statistical comparisons demonstrated significant differences in mutation rates between the four-year post-vaccination and post-third vaccination time points in both group H (P = 0.009) and group L (P = 0.014). Although inter-group comparisons did not reach statistical significance, group H consistently exhibited higher mutation frequencies compared to group L following the third vaccination (**Figure 4A**).

Analysis of the number of mutated amino acids in 1000 amino acids of the V gene segments revealed that the number of mutated amino acids in the IgG-H CDR3 repertoires of both the group H and the group L was mainly concentrated in the “0-100” range. Statistical analysis showed that in the group H, the number of mutated amino acids in the ranges “101-150” (P = 0.028), “151-200” (P = 0.009), and “>200” (P = 0.009) significantly increased after the third vaccination compared to after the second vaccination. Four years post-vaccination, the number of mutated amino acids in the ranges “51-100” (P = 0.016), “101-150” (P = 0.016), “151-200” (P = 0.009), and “>200” (P = 0.028) significantly increased compared to after the third vaccination. In the group L, the number of mutated amino acids in the ranges “151-200” and “>200” also increased after the third vaccination compared to after the second vaccination, with only the range “151-200” showing a statistically significant difference (P = 0.028) (**Figure 4B**).

The CDR3 region is located at the junction between the *V*(*D*)*J* gene segments. During the formation of gene segments, nucleotides are inserted and deleted. In addition to the combinatorial diversity and SHM mentioned above, this junctional diversity also contributes to the diversity of the CDR3 repertoire. Analysis of the average number of nucleotide insertions and deletions per sample in the group H and the group L revealed no significant differences at any time point (**Figure 4C**).

### Analysis of IgG-H CDR3 repertoire overlap

**Figure 5:**
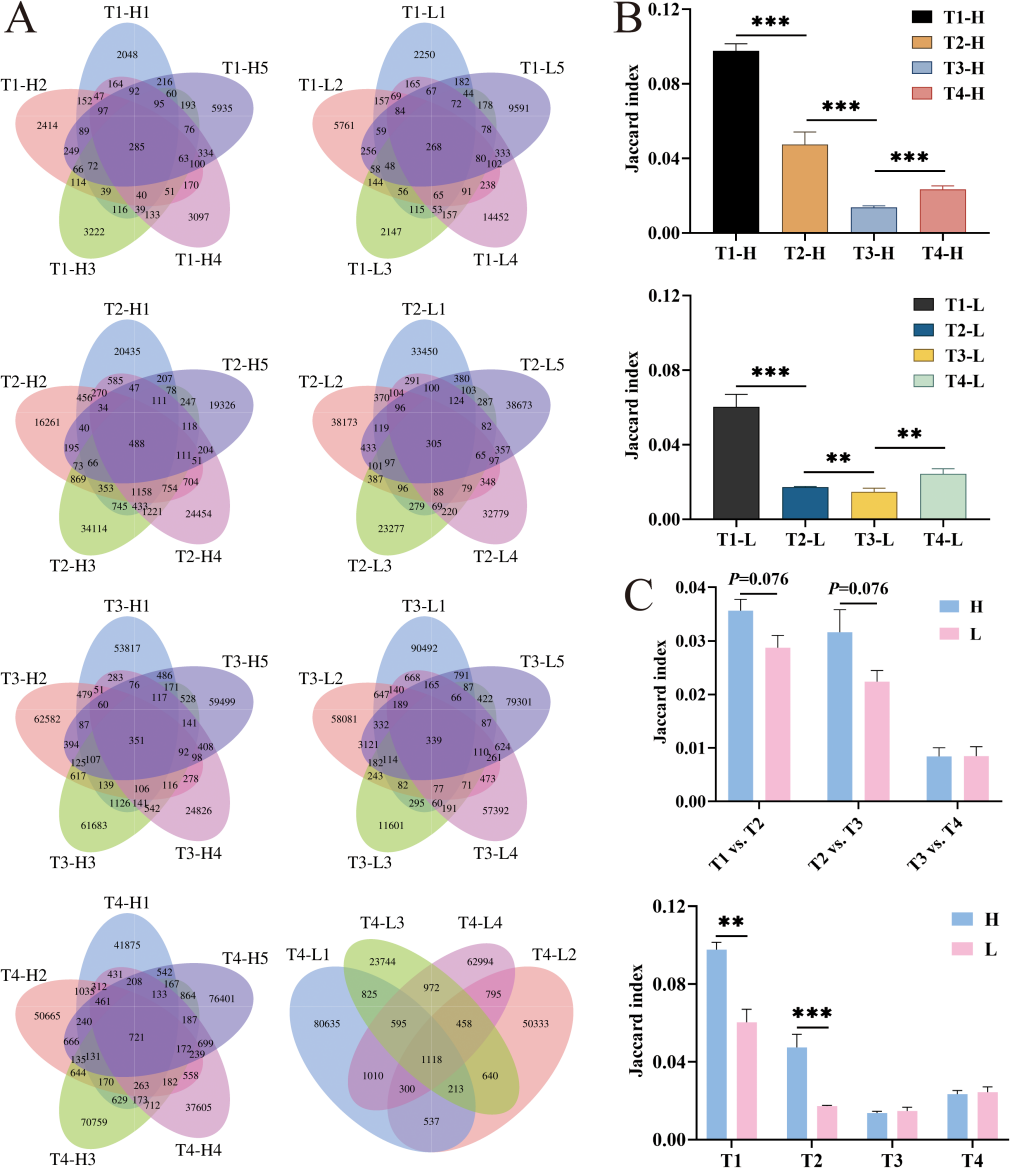
The overlap number and the Jaccard index analysis of IgG-H CDR3 repertories in group H and group L at T1, T2, T3 and T4. **(A)** The overlap number in group H and group L at four blood collection time points; **(B)** The Jaccard index in group H and group L; **(C)** Jaccard index of overlapping clusters between adjacent time points in group H and group L. Note. In the figure **(A)**, the left part shows results for group H and the right part for group L. In the figure **(B)**, the top part shows results for group H and the bottom part for group L. (Mann-Whitney U test, *P < 0.05, **P < 0.01, ***P < 0.001).

To quantitatively assess the temporal dynamics of immune repertoire overlap, we performed a comprehensive analysis of unique cluster sharing between group H and group L across four distinct time points: pre-vaccination, post-second vaccination, post-third vaccination, and the four-year post-vaccination time point. The degree of repertoire overlap was quantified using the Jaccard similarity coefficient (28, 29), a well-established metric in set comparison analysis. The coefficient was calculated according to the following formula: J(A, B) = |A∩B|/|A∪B|, where A and B represent the unique cluster sets from two different conditions. This index ranges from 0 to 1, with values approaching 1 indicating a higher degree of similarity between the compared datasets, reflecting an increased proportion of shared clusters relative to the total cluster population. The results showed that the overlap rate significantly decreased in both the group H (*P* < 0.001) and the group L (*P* < 0.001) after the second vaccination, with a more pronounced decrease in the group H (*P* < 0.001) compared to the group L (P = 0.002) after the third vaccination. Additionally, comparing the Jaccard indices between the group H and the group L revealed that the group H had significantly higher Jaccard indices than the group L at pre-vaccination (P = 0.002) and post-second vaccination (P < 0.001). Calculation of the Jaccard indices between pairs of time points in the group H and the group L showed that the overlap rates in the group H were higher than those in the group L between pre-vaccination and post-second vaccination (P = 0.076) and between post-second vaccination and post-third vaccination (P = 0.076), but these differences were not statistically significant (**Figure 5**).

Analysis of overlapping clusters was conducted by screening clusters that overlapped between the second and third vaccinations, ranked within the top 200 in frequency, and had a count of less than 10 before vaccination. These clusters were defined as HBV vaccine-related clusters. Such clusters were identified in all five samples of the group H. When these related clusters were searched for in the clusters four years post-vaccination, it was found that some clusters had disappeared, while others persisted at lower frequencies (**Supplementary Tables S4**-**S8**). When the same screening criteria were applied to the group L, it was observed that most sequences in the group L exhibited higher counts before vaccination and after the third vaccination, with no significant clonal expansion after the second vaccination. Searching for these screened clusters in the clusters four years post-vaccination also revealed that some clusters had disappeared, while most others persisted at lower frequencies (**Supplementary Tables S9**-**S13**).

The sequences contained in the related clusters screened from the group H in this study were compared with HBV-related sequences reported in the literature (11–13, 30–36) (**Supplementary Table S14**). Although no identical sequences were found, common conserved motifs such as “YGLDV”, “DAFD”, “YGSGS”, “GAFDI”, and “NWFDP” (underlined in the table) were identified in multiple samples of the group H, which are associated with HBV-related sequences.

## Discussion

In accordance with the vaccine recipient monitoring protocol stipulated in the “Guidelines for the Prevention and Treatment of Chronic Hepatitis B (2022 Edition),” this study focuses on longitudinal monitoring of HBsAb titer dynamics. The remaining four serological markers consistently maintained negative status post-vaccination, thus obviating the need for repeated testing (37). In this study, among 49 HBV vaccination volunteers, five volunteers with ultra-high HBsAb levels and five volunteers with extremely low HBsAb levels were screened by monitoring HBsAb levels after the first dose. HBsAb levels were continuously monitored after the third dose and four years post-vaccination. It was found that the five volunteers with ultra-high HBsAb levels maintained ultra-high HBsAb levels in their peripheral blood (with only one volunteer dropping below 1000 mIU/mL after four years). The HBsAb levels peaked after the second booster dose, suggesting that a large number of memory B cells were involved in the response after the second dose. A consistent downward trend in HBsAb levels was observed after the third dose and four years later. Among the five volunteers with extremely low baseline HBsAb levels (all <10 mIU/mL pre-vaccination), three post-vaccination monitoring timepoints consistently showed levels below 100 mIU/mL. Notably, one volunteer transiently exceeded 100 mIU/mL after the third dose but declined below this threshold by the 4-year follow-up. In Group L, None of the 5 volunteers achieved protective HBsAb levels (≥10 mIU/mL) after the second dose. Only a subset maintained low-level protection (10–100 mIU/mL) after the third dose and four years post-vaccination. This suggests that further exploration of the special mechanisms of B cell responses to HBV vaccination can be conducted using volunteers with ultra-high and very low HBsAb levels.

With the development and application of high-throughput sequencing (HTS) technology for assessing individual BCR repertoires, changes in the BCR CDR3 repertoire of B cells after HBV vaccination can be analyzed at the genetic level to evaluate the body’s response to the HBV vaccine. In this study, the diversity of each sample was analyzed by calculating the inverse Simpson index. The diversity in the group H decreased after the second vaccination and increased after the third vaccination, both showing statistical differences compared to pre-vaccination. The group L also exhibited a decrease in diversity after the second vaccination and an increase after the third vaccination, but the differences were not significant. This result is consistent with the findings of Miyasaka et al. (14). The decrease in diversity after the second vaccination indicates clonal expansion of B cells post-vaccination, with the group H showing more significant clonal expansion than the group L. This suggests that the large differences in HBsAb levels after HBV vaccination may be related to the sequences of high-frequency B cell clones. Additionally, the diversity of the BCR-H CDR3 repertoire can serve as a preliminary assessment indicator for populations with different HBsAb levels.

In studies of BCR CDR3 repertoires following vaccination or infection, the analysis of *IGHV* and *IGHJ* gene usage and pairing has consistently garnered attention (12–14, 16, 36). The results of this study show that, compared to the group L, the group H exhibited higher post-vaccination usage frequencies of *IGHV1-18*, *IGHV2-26*, *IGHV2-5*, *IGHV3-21*, *IGHV3-23*, *IGHV3-33*, *IGHV3-7*, *IGHV3-74*, *IGHV3-9*, and *IGHV7–27* compared to pre-vaccination. In contrast, the usage of *IGHJ* genes showed no significant differences, with both the group H and the group L frequently using *IGHJ4*. The dominant *IGHV* and *IGHJ* genes identified in this study are consistent with findings from related research. For instance, *IGHV3–7* has been reported to significantly increase after HBV vaccination (13), and our previous research also found increased usage of *IGHV3–23* in volunteers with ultra-high HBsAb levels (15). Additionally, Jiang et al. (36) reported increased usage of *IGHV3–30* and *IGHV4–59* after HBV vaccination. In this study, the usage of *IGHV3–30* was relatively low, while *IGHV4–59* was frequently utilized in both the group H and the group L. In the analysis of *IGHV-IGHJ* pairings, the frequencies of *IGHV1-18-IGHJ4*, *IGHV4-4-IGHJ4*, and *IGHV6-1-IGHJ4* pairings were significantly higher in the group H than in the group L. Among these, the dominant pairing of *IGHV4-34-IGHJ4* aligns with the findings of our previous research (15). These studies suggest that dominant gene usage and gene pairings may be related to vaccine responses, providing a foundation for further exploration of HBV vaccine responses. Vaccine Response Prediction: Proposed collection of large-scale samples from vaccinated cohorts, quantitative measurement of *IGHV1-18/IGHJ4* expression levels, integration with longitudinal immunogenicity data (anti-HBs titers, cellular responses), development of machine learning models for response prediction, rigorous validation of predictive performance metrics. Clinical Diagnostics & Prognostics: Systematic evaluation of *IGHV1-18/IGHJ4* expression patterns across.

In this study, the CDR3 length distribution in both the group H and the group L followed a normal distribution. Existing research reports indicate that the IgG-H CDR3 repertoire, regardless of individual status, tends to have similar length distributions (30). Analysis of amino acid usage in this study revealed a preference for hydrophilic amino acids during the body’s response to HBV vaccination, a finding consistent with most current literature. Whether in cases of HBV infection or HBV vaccination, and regardless of HBsAb levels, amino acid usage patterns are similar (8, 29, 38).

Somatic hypermutation is a key mechanism for diversifying the BCR CDR3 repertoire during the germinal center reaction, followed by the selection of high-affinity mutants leading to “affinity maturation,” which may result in the accumulation of memory B cells. This study first analyzed the average mutation rates in the group H and the group L at different time points. The results showed that, except for a decrease four years post-vaccination, the average mutation rates were similar to those before vaccination. However, comparing the average mutation rates between the group H and the group L at different time points revealed that the average mutation rate in the group H was slightly higher than that in the group L after the third vaccination. Further analysis of the number of mutated amino acids in 1000 amino acids showed that the group H had a significant increase in the number of mutations in the ranges “151-200” and “>200” after the third vaccination, while the group L only showed a significant increase in the range “151-200.” This indicates that the group H underwent more mutations than the group L after the third vaccination, suggesting that individuals with ultra-high HBsAb levels achieved greater affinity maturation after completing the full course of HBV vaccination compared to those with lower HBsAb levels. Zhao et al. (10) investigated the development of memory B cells into plasma cells after HBV vaccination by analyzing somatic hypermutation. In their study, memory B cell clones were considered somatic mutants of plasma cell clones, indicating that clones in memory B cells developing into plasma cells underwent stronger somatic hypermutation. This provides a solid foundation for further exploration of the mechanisms underlying ultra-high HBsAb levels. However, no significant differences were observed in the average number of nucleotide insertions and deletions between the group H and the group L. Hong et al. (38) found that the incidence of the 5’ end of the D gene (5DP) in the IgG-H CDR3 repertoire of chronic HBV carriers was higher than that in healthy volunteers, but its contribution to increasing clonal diversity was minimal because the occurrence rate of 5DP itself was lower than other types of junctional modifications in the CDR3 region. This suggests that insertions and deletions may not be the primary cause of diversity differences between the group H and the group L.

In further comparative analysis, we made an innovative discovery: we identified multiple common conserved “motifs” in the group H, such as “YGLDV,” “DAFD,” “YGSGS,” “GAFDI,” and “NWFDP,” which are also found in HBV-related sequences reported in the literature. Currently, few studies have reported common “motifs” in the BCR CDR3 repertoires of individuals with different HBsAb levels after HBV vaccination. Yan et al. (39) found common “GGETQ” or “GETQ” motifs in the TCR CDR3 repertoires of individuals who responded to HBV vaccination. Zhan et al. (40) also identified the “GAGPLT” motif exclusively in healthy individuals with isolated HBsAb positivity, suggesting its potential association with HBsAb production/maintenance. The shared “motif” identified through screening is believed to be a vaccination-specific motif associated with antibody production. These common “motifs” help reveal the mechanisms underlying the production of different HBsAb levels after HBV vaccination and serve as potential targets for antibody affinity research. They also provide a foundation for optimizing the design of future hepatitis B vaccines and for developing technologies to prepare individuals with ultra-high HBsAb levels after HBV vaccination, as well as for the prevention and treatment of HBV infection. Although this study observed the enrichment of known anti-HBV-associated motifs such as ‘YGLDV’, these findings only suggest potential antigen-driven selection. The specific binding capability of these motifs to HBsAg still requires further validation through *in vitro* binding assays (e.g., ELISA) or subsequent studies involving single-cell antibody cloning and expression.

The innovation of this study lies in addressing the key scientific question of differential HBsAb levels in populations post-HBV vaccination. We specifically selected volunteers demonstrating either ultra-high or extremely low HBsAb responses following HBV vaccination as our study subjects. Utilizing the Illumina HiSeq X Ten sequencing platform with HTS technology and analytical systems, we conducted comprehensive comparative analyses of: (1) dynamic variations in HBsAb levels, and (2) compositional characteristics of IgG-H CDR3 repertoires between these two extreme response groups. This investigation aims to elucidate the potential mechanisms underlying polarized HBsAb production (ultra-high vs. extremely low) following HBV vaccination, thereby providing fundamental data and novel research perspectives for: Developing vaccination strategies to enhance HBsAb production and improving prevention and control measures against HBV infection.

This study has several important limitations that should be acknowledged. First, the statistical power was limited by the relatively small sample size (n=5 per group). Although we employed an extreme phenotype design to mitigate this constraint, the generalizability of our findings requires verification through larger-scale studies. Additionally, potential confounding effects of sex and age were not considered in the current study, but will be included as covariates in subsequent analyses. Second, this study encountered a vaccine supply limitation at participating clinical sites: participants received GlaxoSmithKline (GSK) vaccines for the first two doses but were administered Dalian Hissen’s vaccine for the third dose due to temporary unavailability of the GSK product. Importantly, both vaccines contained recombinant HBsAg expressed through identical production systems and exhibited matching antigenic epitope profiles. This molecular consistency suggests comparable immunogenicity, with no expected divergence in B-cell mediated immune responses between vaccine products. Finally, in the current study, we did not perform direct binding validation (e.g., surface plasmon resonance, SPR) or functionally characterize antibodies encoded by the enriched motifs. In future studies, we plan to: Conduct single-cell sequencing of HBsAg-specific B cells. Express recombinant antibodies based on their native H-L chain pairing. Perform comprehensive functional characterization (affinity measurements and neutralization assays). These investigations will provide foundational data for developing therapeutic HBsAbs.

## Conclusion

This comprehensive study employed a detailed comparative analysis of ultra-high responders and extremely low responders to HBV vaccination, revealing that elevated HBsAb levels in ultra-high responders were consistently maintained throughout the four-year monitoring period. Through systematic comparison of IgG-H CDR3 repertoire composition across multiple time points between these two distinct response groups, we identified potential mechanistic associations with characteristic *IGHV* gene usage patterns and somatic hypermutation frequencies. These findings provide novel insights into the complex immunological mechanisms underlying HBV vaccine responses and HBV infection dynamics. Furthermore, this study establishes an innovative methodological framework for the identification and production of high-affinity, high-titer HBsAb through vaccine-induced immune responses, potentially advancing the development of therapeutic antibodies and vaccine optimization strategies.

## Data Availability

The datasets presented in this study can be found in online repositories. The names of the repository/repositories and accession number(s) can be found in the article/**Supplementary Material**.
