# Supplementary material for: Dynamics of HBsAb persistence and associated BCR IgG-H CDR3 repertoire features in ultra-high versus extremely low responders to HBV vaccination
Source: Front Immunol. 2025 Jul 4;16:1587553. doi: 10.3389/fimmu.2025.1587553 (PMC12271205; doi:10.3389/fimmu.2025.1587553)
Supplement: Supplementary file 1 [file Table1.docx]

**Supplementary table 1 Main Reagents**

| **Main Reagents** | **Origin** | **Company** |
| --- | --- | --- |
| GE Ficoll-Paque™ PLUS lymphocyte separation medium | USA | GE Healthcare |
| Red Blood Cell Lysis Buffer | China | Tianjin Haoyang Biological Products Technology Co., Ltd. |
| Phosphate Buffered Saline (PBS) | China | Beijing Solarbio Science & Technology Co., Ltd. |
| RNeasy Plus Mini Kit | Germany | QIAGEN |
| TRIzol™ Reagent | USA | Thermo Fisher Scientific |
| Absolute Ethanol | China | Chengdu Jinshan Chemical Reagent Co., Ltd. |
| Isopropanol | China | Chengdu Jinshan Chemical Reagent Co., Ltd. |
| Chloroform | China | Chongqing Chuandong Chemical (Group) Co., Ltd. |
| DEPC-treated Water | China | Shanghai Jierui Biological Engineering Co., Ltd. |
| 50×TAE Buffer | China | Beijing Solarbio Science & Technology Co., Ltd. |
| GoldView™ Nucleic Acid Stain (Type I) | China | Beijing Solarbio Science & Technology Co., Ltd. |
| RevertAid First Strand cDNA Synthesis Kit | USA | Thermo Fisher Scientific |
| DNA Marker | Japan | TaKaRa |
| Gel Extraction Kit | Germany | QIAGEN |
| Taq PCR Master Mix Kit | Germany | QIAGEN |
| Hepatitis B Virus Surface Antigen (HBsAg) ELISA Kit | China | Beijing Wantai Biological Pharmacy Enterprise Co., Ltd. |
| Hepatitis B Virus Surface Antibody (HBsAb) ELISA Kit | China | Beijing Wantai Biological Pharmacy Enterprise Co., Ltd. |
| Hepatitis B Virus e-Antigen (HBeAg) ELISA Kit | China | Beijing Wantai Biological Pharmacy Enterprise Co., Ltd. |
| Hepatitis B Virus e-Antibody (HBeAb) ELISA Kit | China | Beijing Wantai Biological Pharmacy Enterprise Co., Ltd. |
| Hepatitis B Virus Core Antibody (HBcAb) ELISA Kit | China | Beijing Wantai Biological Pharmacy Enterprise Co., Ltd. |

**Supplementary table 2 HBsAb levels of 45 volunteers**

| <10 mIU/mL | 10-99 mIU/mL | 100-999 mIU/mL | 1000-9999 mIU/mL | >10000 mIU/mL |
| --- | --- | --- | --- | --- |
| 5.82 | 94.28 | 897.3 | 9958.96 | 61253.73 |
| 5.06 | 67.63 | 603.14 | 8730.09 | 39854.57 |
| 2.78 | 53.2 | 485.6 | 8712.45 | 17896.48 |
| 1.15 | 30.48 | 395.67 | 6808.25 | 16920.24 |
| 0.93 | 11.36 | 227.81 | 6722.95 | 16569.18 |
|  | 10.44 | 114.68 | 6647.1 | 14130.51 |
|  | 10.36 | 111.3 | 6628.58 | 11537.96 |
|  |  |  | 6304.4 | 10853.62 |
|  |  |  | 5540.4 |  |
|  |  |  | 4497.05 |  |
|  |  |  | 4348.72 |  |
|  |  |  | 3742.19 |  |
|  |  |  | 2210.97 |  |
|  |  |  | 2168.01 |  |
|  |  |  | 1929.7 |  |
|  |  |  | 1654.96 |  |
|  |  |  | 1464.73 |  |
|  |  |  | 1288.46 |  |

**Supplementary table 3 HTS sequencing data of IgG-H CDR3 repertoires**

| **Sample ID** | **Raw Sequence**^a^ | **Analysis Sequence Count**^b^ | **Analysis Sequence Clonotype**^b^ | **Cluster**^c^ |
| --- | --- | --- | --- | --- |
| T1-H1 | 739909 | 11084 | 4260 | 3651 |
| T1-H2 | 775443 | 21142 | 5224 | 4048 |
| T1-H3 | 938814 | 29244 | 6122 | 4664 |
| T1-H4 | 964757 | 34126 | 6324 | 4883 |
| T1-H5 | 1116563 | 93050 | 11760 | 8022 |
| T2-H1 | 1744428 | 433763 | 36422 | 25506 |
| T2-H2 | 1463131 | 305806 | 31366 | 21883 |
| T2-H3 | 2144185 | 676361 | 58075 | 40939 |
| T2-H4 | 1822453 | 370213 | 42108 | 30743 |
| T2-H5 | 1428908 | 342665 | 28137 | 21396 |
| T3-H1 | 2433015 | 696409 | 70781 | 57597 |
| T3-H2 | 2735343 | 937807 | 823216 | 65682 |
| T3-H3 | 3278662 | 1442543 | 93550 | 66102 |
| T3-H4 | 2108888 | 540490 | 38156 | 27686 |
| T3-H5 | 2888814 | 1025973 | 83348 | 62740 |
| T4-H1 | 7352551 | 544431 | 51566 | 47491 |
| T4-H2 | 6313663 | 774916 | 63089 | 56594 |
| T4-H3 | 5340543 | 1131797 | 85936 | 76042 |
| T4-H4 | 6258038 | 472468 | 48002 | 43056 |
| T4-H5 | 7178695 | 1591758 | 91527 | 81966 |
| T1-L1 | 862505 | 14921 | 4554 | 3754 |
| T1-L2 | 1281371 | 77839 | 10508 | 7536 |
| T1-L3 | 980199 | 21530 | 4528 | 3654 |
| T1-L4 | 1685445 | 253801 | 21719 | 16374 |
| T1-L5 | 1016263 | 125888 | 16259 | 11500 |
| T2-L1 | 2138115 | 486800 | 46880 | 36071 |
| T2-L2 | 2334625 | 925601 | 64145 | 40958 |
| T2-L3 | 1738829 | 284641 | 32677 | 25659 |
| T2-L4 | 1859812 | 440566 | 43354 | 35204 |
| T2-L5 | 2138352 | 784603 | 57072 | 41419 |
| T3-L1 | 3722890 | 1234863 | 118040 | 94544 |
| T3-L2 | 2836605 | 745294 | 80471 | 64462 |
| T3-L3 | 1355254 | 195379 | 18296 | 14027 |
| T3-L4 | 3233224 | 843006 | 76708 | 60913 |
| T3-L5 | 3206037 | 1490930 | 111766 | 86191 |
| T4-L1 | 6840538 | 2317381 | 96733 | 85233 |
| T4-L2 | 4482048 | 840541 | 60128 | 54394 |
| T4-L3 | 5246948 | 304195 | 32971 | 28565 |
| T4-L4 | 6820568 | 1095332 | 77062 | 68242 |

Note: (a) The total number of raw sequences obtained after HTS sequencing by BGI; (b) The Count and Clonotype numbers of sequences that can be analyzed after comparison with NCBI;( c) The Cluster obtained after clustering.

**Supplementary table 4 Characterization of specific clusters of sample H1 of IgG-H CDR3 repertories at T1, T2, T3 and T4**

| ID | *IGHV* | *IGHJ* | CDR3 sequence | T1 | T2 | T3 | T4 |
| --- | --- | --- | --- | --- | --- | --- | --- |
| 1 | *IGHV5-51* | *IGHJ3* | CARPLIGDSKGIEIW | 8  (0.072%) | 1999  (0.461%) | 6400  (0.919%) | / |
| 2 | *IGHV4-55* | *IGHJ5* | CARSHAGPW | 2  (0.018%) | 2943  (0.678%) | 6069  (0.871%) | / |
| 3 | *IGHV4-39* | *IGHJ5* | CARSPAGEKLNWFDPW | 2  (0.018%) | 1137  (0.262%) | 5914  (0.849%) | / |
| 4 | *IGHV4-4* | *IGHJ6* | CARMPIVAARGYGMDVW | 4  (0.036%) | 1156  (0.267%) | 4266  (0.613%) | / |
| 5 | *IGHV4-39* | *IGHJ4* | CARHPKYCSATSCRGGGLDSW | 3  (0.027%) | 440  (0.101%) | 3798  (0.545%) | / |
| 6 | *IGHV5-51* | *IGHJ3* | CVRPTVGDSHDGVDMW | 4  (0.036%) | 1103  (0.254%) | 3104  (0.446%) | / |
| 7 | *IGHV5-51* | *IGHJ4* | CARHPAPKGRLDYW | 6  (0.054%) | 1270  (0.293%) | 2460  (0.353%) | / |
| 8 | *IGHV4-39* | *IGHJ3* | CARHAERTDLIPLVSDAFDIW | 3  (0.027%) | 301  (0.069%) | 2301  (0.330%) | 11  (0.002%) |
| 9 | *IGHV4-34* | *IGHJ5* | CARSPAGEQLGWFDPW | 2  (0.018%) | 544  (0.125%) | 1848  (0.265%) | / |
| 10 | *IGHV4-39* | *IGHJ4* | CARLGYGSGSYFDYW | 2  (0.018%) | 422  (0.097%) | 1706  (0.245%) | 14  (0.003%) |
| 11 | *IGHV4-39* | *IGHJ6* | CTRRKYYDPVGMDVW | 1  (0.009%) | 393  (0.091%) | 1701  (0.244%) | / |
| 12 | *IGHV5-51* | *IGHJ4* | CARPATSYTSSSHFDSW | 3  (0.027%) | 530  (0.122%) | 1627  (0.234%) | 31  (0.006%) |
| 13 | *IGHV5-51* | *IGHJ3* | CARLRYGAGTYRAVDLW | 2  (0.018%) | 727  (0.168%) | 1577  (0.226%) | 4  (0.001%) |
| 14 | *IGHV5-51* | *IGHJ5* | CVRLNIVTTMGLANWFDPW | 8  (0.072%) | 321  (0.074%) | 1415  (0.203%) | / |
| 15 | *IGHV4-4* | *IGHJ6* | CTRRKYYDPVGMDVW | 1  (0.009%) | 420  (0.097%) | 1373  (0.197%) | 5  (0.001%) |
| 16 | *IGHV4-39* | *IGHJ6* | CARQKEPDRYYYYGLDVW | 2  (0.018%) | 844  (0.195%) | 1299  (0.187%) | / |
| 17 | *IGHV4-39* | *IGHJ4* | CARQRLTGSAGRGFCEYW | 4  (0.036%) | 345  (0.080%) | 1038  (0.149%) | 18  (0.003%) |
| 18 | *IGHV6-1* | *IGHJ5* | CARDFAPTGSRWFDVW | 2  (0.018%) | 353  (0.081%) | 983  (0.141%) | 54  (0.010%) |
| 19 | *IGHV1-18* | *IGHJ4* | CVRGAWGQHIDSW | 2  (0.018%) | 340  (0.078%) | 947  (0.136%) | / |
| 20 | *IGHV4-39* | *IGHJ2* | CARRMVMISNWYFDPW | 2  (0.018%) | 611  (0.141%) | 943  (0.135%) | / |
| 21 | *IGHV5-51* | *IGHJ6* | CARRSGESSRFSRMDVW | 6  (0.054%) | 301  (0.069%) | 899  (0.129%) | / |
| 22 | *IGHV1-18* | *IGHJ4* | CARDTWFGDLSSLAYW | 3  (0.027%) | 302  (0.070%) | 892  (0.128%) | 21  (0.004%) |
| 23 | *IGHV5-51* | *IGHJ5* | CARLFGEQWLIPDYW | 2  (0.018%) | 655  (0.151%) | 855  (0.123%) | / |
| 24 | *IGHV4-4* | *IGHJ4* | CAREGGCYGPW | 2  (0.018%) | 360  (0.083%) | 830  (0.119%) | / |
| 25 | *IGHV4-39* | *IGHJ4* | CARRRAEAGMYDHW | 2  (0.018%) | 297  (0.068%) | 621  (0.089%) | / |
| 26 | *IGHV5-51* | *IGHJ4* | CARRLDSGSDFW | 2  (0.018%) | 250  (0.058%) | 562  (0.081%) | / |
| 27 | *IGHV6-1* | *IGHJ4* | CARDPPDDQGLDYW | 1  (0.009%) | 329  (0.076%) | 530  (0.076%) | / |
| 28 | *IGHV4-4* | *IGHJ3* | CARDGGDSGTPPLRSFDIW | / | 987  (0.228%) | 5936  (0.852%) | 70  (0.013%) |
| 29 | *IGHV4-59* | *IGHJ3* | CARIKGSGSAGDMW | / | 1609  (0.371%) | 5474  (0.786%) | / |
| 30 | *IGHV6-1* | *IGHJ3* | CARSGALEKGLADAFDIW | / | 619  (0.143%) | 4167  (0.598%) | 81  (0.015%) |
| 31 | *IGHV5-51* | *IGHJ5* | CARGNVENWFGPW | / | 992  (0.229%) | 2650  (0.381%) | / |
| 32 | *IGHV4-4* | *IGHJ5* | CARHRGGYSSFDSW | / | 508  (0.117%) | 2124  (0.305%) | / |
| 33 | *IGHV4-39* | *IGHJ6* | CARQHNWNYTPHNYGMDVW | / | 797  (0.184%) | 2098  (0.301%) | / |
| 34 | *IGHV4-39* | *IGHJ6* | CARHFTSSRPAGMDVW | / | 1779  (0.410%) | 2028  (0.291%) | / |
| 35 | *IGHV5-51* | *IGHJ4* | CVRRGGDYFRSLYYFDYW | / | 263  (0.061%) | 1240  (0.178%) | / |
| 36 | *IGHV4-39* | *IGHJ4* | CARHAIFGVVHVDYW | / | 287  (0.066%) | 1225  (0.176%) | / |
| 37 | *IGHV4-39* | *IGHJ5* | CSRHPAAEQLGWFDPW | / | 269  (0.062%) | 1183  (0.170%) | / |
| 38 | *IGHV4-4* | *IGHJ6* | CARDVTFEGVTTRGFDVW | / | 376  (0.087%) | 1074  (0.154%) | / |
| 39 | *IGHV4-59* | *IGHJ4* | CARARGIVATPFDYW | / | 350  (0.081%) | 1035  (0.149%) | / |
| 40 | *IGHV4-39* | *IGHJ5* | CARHGHSSRFDPW | / | 254  (0.059%) | 899  (0.129%) | / |
| 41 | *IGHV6-1* | *IGHJ3* | CVRVTWESRASDVW | / | 307  (0.071%) | 856  (0.123%) | / |
| 42 | *IGHV4-39* | *IGHJ5* | CARQPSKVTLTEW | / | 512  (0.118%) | 830  (0.119%) | / |
| 43 | *IGHV5-51* | *IGHJ4* | CARHKEPLVDYW | / | 540  (0.124%) | 825  (0.118%) | / |
| 44 | *IGHV5-51* | *IGHJ4* | CARRVFDDLTGSAYFDSW | / | 286  (0.066%) | 816  (0.117%) | / |
| 45 | *IGHV4-59* | *IGHJ3* | CAKEGSVADRNAFHFW | / | 305  (0.070%) | 759  (0.109%) | / |
| 46 | *IGHV4-59* | *IGHJ4* | CARRWSGVDYW | / | 636  (0.147%) | 733  (0.105%) | / |
| 47 | *IGHV5-51* | *IGHJ4* | CARALNGLFYFDYL | / | 304  (0.070%) | 733  (0.105%) | / |
| 48 | *IGHV4-39* | *IGHJ6* | CGRLSDFYGLDVW | / | 262  (0.060%) | 640  (0.092%) | / |
| 49 | *IGHV4-4* | *IGHJ4* | CARDYSAWTRLEYW | / | 1471  (0.339%) | 639  (0.092%) | / |
| 50 | *IGHV5-51* | *IGHJ4* | CARPGNSIWYYFDHW | / | 278  (0.064%) | 568  (0.082%) | / |
| 51 | *IGHV5-51* | *IGHJ4* | CARIYERPRGLDYW | / | 266  (0.061%) | 548  (0.079%) | 1  (0.0002%) |
| 52 | *IGHV4-39* | *IGHJ4* | CARGHGGYW | / | 2846  (0.656%) | 530  (0.076%) | / |
| 53 | *IGHV1-2* | *IGHJ5* | CSSDPTGNWFDPW | / | 326  (0.075%) | 508  (0.073%) | / |
| 54 | *IGHV4-4* | *IGHJ3* | CVRAWGSGGSNEEHALDVW | / | 324  (0.075%) | 489  (0.070%) | / |

**Supplementary table 5 Characterization of specific clusters of sample H2 of IgG-H CDR3 repertories at T1, T2, T3 and T4**

| ID | *IGHV* | *IGHJ* | CDR3 sequence | T1 | T2 | T3 | T4 |
| --- | --- | --- | --- | --- | --- | --- | --- |
| 1 | *IGHV4-4* | *IGHJ3* | CARHLGHTSSWFDAFDIW | 2  (0.010%) | 1064  (0.348%) | 25736  (2.744%) | / |
| 2 | *IGHV4-39* | *IGHJ4* | CARHRAWTNCYLDSW | 4  (0.019%) | 579  (0.189%) | 6534  (0.697%) | / |
| 3 | *IGHV4-34* | *IGHJ5* | CARGKQLWFQSPPHNWFDPW | 4  (0.019%) | 235  (0.077%) | 4386  (0.468%) | 1  (0.0001%) |
| 4 | *IGHV6-1* | *IGHJ5* | CASGLSSIGFDPW | 4  (0.019%) | 190  (0.062%) | 2984  (0.318%) | / |
| 5 | *IGHV5-51* | *IGHJ4* | CARLVGPEQYDRSGYLDYW | 2  (0.010%) | 184  (0.060%) | 2631  (0.281%) | / |
| 6 | *IGHV5-51* | *IGHJ4* | CARLFGPDHYDASGYLDYW | / | 217  (0.071%) | 4559  (0.486%) | / |
| 7 | *IGHV4-59* | *IGHJ4* | CARDREHTHGRHFGYW | / | 322  (0.105%) | 4454  (0.475%) | 269  (0.035%) |

**Supplementary table 6 Characterization of specific clusters of sample H3 of IgG-H CDR3 repertories at T1, T2, T3 and T4**

| ID | *IGHV* | *IGHJ* | CDR3 sequence | T1 | T2 | T3 | T4 |
| --- | --- | --- | --- | --- | --- | --- | --- |
| 1 | *IGHV1-18* | *IGHJ4* | CARIPPNRGRSSGWYVDYW | 3  (0.010%) | 462  (0.068%) | 18193  (1.261%) | / |
| 2 | *IGHV4-4* | *IGHJ4* | CARERSGFSVNFDYW | 8  (0.027%) | 1267  (0.187%) | 17635  (1.222%) | / |
| 3 | *IGHV4-39* | *IGHJ3* | CARPSTIATRAGAFDIW | 8  (0.027%) | 697  (0.103%) | 15521  (1.076%) | / |
| 4 | *IGHV4-39* | *IGHJ6* | CARHEAGWLIVGRPKYGMDVW | 6  (0.021%) | 483  (0.071%) | 14580  (1.010%) | / |
| 5 | *IGHV6-1* | *IGHJ6* | CARANLLGRGMNVW | 8  (0.027%) | 763  (0.113%) | 9574  (0.664%) | / |
| 6 | *IGHV5-51* | *IGHJ3* | CARRAYDYDLLTGGVDAFDIW | 5  (0.017%) | 588  (0.087%) | 3321  (0.230%) | / |
| 7 | *IGHV5-51* | *IGHJ6* | CVRIYPTNGLRYPRGVGYGLDVW | 2  (0.007%) | 722  (0.107%) | 2651  (0.184%) | 1278  (0.113%) |
| 8 | *IGHV4-28* | *IGHJ4* | CARSWPNWLFPFDSW | 2  (0.007%) | 2363  (0.349%) | 2135  (0.148%) | / |
| 9 | *IGHV5-51* | *IGHJ4* | CATIAHPLGILYW | 2  (0.007%) | 1535  (0.227%) | 1973  (0.137%) | 4  (0.0004%) |
| 10 | *IGHV4-4* | *IGHJ5* | CASGTDPYKTGHW | / | 1451  (0.215%) | 4374  (0.303%) | / |
| 11 | *IGHV6-1* | *IGHJ4* | CARWGGPAAGFDYW | / | 1735  (0.257%) | 2281  (0.158%) | 3  (0.0003%) |
| 12 | *IGHV4-4* | *IGHJ4* | CARDPGYGSGRTDYW | / | 811  (0.120%) | 1671  (0.116%) | / |

**Supplementary table 7 Characterization of specific clusters of sample H4 of IgG-H CDR3 repertories at T1, T2, T3 and T4**

| ID | *IGHV* | *IGHJ* | CDR3 sequence | T1 | T2 | T3 | T4 |
| --- | --- | --- | --- | --- | --- | --- | --- |
| 1 | *IGHV5-51* | *IGHJ3* | CVRPTVGDSHDGVDMW | 6  (0.018%) | 847  (0.229%) | 538  (0.100%) | / |
| 2 | *IGHV6-1* | *IGHJ5* | CARGYGSGSYDCW | 4  (0.012%) | 329  (0.089%) | 492  (0.091%) | / |
| 3 | *IGHV4-55* | *IGHJ5* | CARSRRVGPTTKGYFDPW | / | 275  (0.074%) | 1139  (0.211%) | / |
| 4 | *IGHV4-4* | *IGHJ4* | CAIPIHIRGYTNW | / | 322  (0.087%) | 613  (0.113%) | / |

**Supplementary table 8 Characterization of specific clusters of sample H5 of IgG-H CDR3 repertories at T1, T2, T3 and T4**

| ID | *IGHV* | *IGHJ* | CDR3 sequence | T1 | T2 | T3 | T4 |
| --- | --- | --- | --- | --- | --- | --- | --- |
| 1 | *IGHV1-18* | *IGHJ4* | CARLVFPGHGDYLSPFDYW | 8  (0.009%) | 1452  (0.424%) | 1137  (0.111%) | 5  (0.0003%) |
| 2 | *IGHV1-18* | *IGHJ4* | CARYLTAAVPGNDYFDHW | / | 660  (0.193%) | 3392  (0.331%) | / |
| 3 | *IGHV1-18* | *IGHJ4* | CARYLTAAVAGKDYFDYW | / | 404  (0.118%) | 1537  (0.150%) | / |
| 4 | *IGHV1-8* | *IGHJ1* | CARGAWPRHEGAEYFHHW | / | 422  (0.123%) | 1002  (0.098%) | / |
| 5 | *IGHV4-39* | *IGHJ5* | CAGLLLWLRVDPW | / | 1368  (0.399%) | 913  (0.089%) | / |

**Supplementary table 9 Characterization of specific clusters of sample L1 of IgG-H CDR3 repertories at T1, T2, T3 and T4**

| ID | *IGHV* | *IGHJ* | CDR3 sequence | T1 | T2 | T3 | T4 |
| --- | --- | --- | --- | --- | --- | --- | --- |
| 1 | *IGHV4-39* | *IGHJ2* | CARLRAGGYSYWYFDLW | 10  (0.067%) | 2  (0.0004%) | 6688  (0.542%) | 10  (0.0004%) |
| 2 | *IGHV5-51* | *IGHJ1* | CARLPYSSSWYRYFQHW | 4  (0.027%) | 9  (0.002%) | 4442  (0.360%) | 454  (0.020%) |
| 3 | *IGHV4-4* | *IGHJ3* | CARREIAMTAFDIW | 2  (0.013%) | 6  (0.001%) | 2697  (0.218%) | 5  (0.0002%) |
| 4 | *IGHV1-2* | *IGHJ4* | CTRKGPTKCFDFW | 1  (0.007%) | 143  (0.029%) | 1924  (0.155%) | 4225  (0.182%) |
| 5 | *IGHV4-4* | *IGHJ3* | CARTNWGGHAFDIW | 2  (0.013%) | 2  (0.0004%) | 1458  (0.118%) | / |
| 6 | *IGHV5-51* | *IGHJ6* | CARQGPPAEDDYYYFALDVW | 2  (0.013%) | 29  (0.006%) | 1192  (0.097%) | 25  (0.001%) |
| 7 | *IGHV4-4* | *IGHJ4* | CARVFCTSTSCPSYFDYW | 2  (0.013%) | 172  (0.035%) | 1131  (0.092%) | 21  (0.001%) |
| 8 | *IGHV4-4* | *IGHJ3* | CAKNMSRTVGGGYWCAFDIW | 2  (0.013%) | 2  (0.0004%) | 955  (0.077%) | / |
| 9 | *IGHV4-34* | *IGHJ4* | CARVSYGSLFEYW | / | 1302  (0.267%) | 1858  (0.150%) | / |
| 10 | *IGHV7-4-1* | *IGHJ6* | CAREPRRLDVW | / | 298  (0.061%) | 1010  (0.082%) | 13  (0.001%) |

**Supplementary table 10 Characterization of specific clusters of sample L2 of IgG-H CDR3 repertories at T1, T2, T3 and T4**

| ID | *IGHV* | *IGHJ* | CDR3 sequence | T1 | T2 | T3 | T4 |
| --- | --- | --- | --- | --- | --- | --- | --- |
| 1 | *IGHV1-18* | *IGHJ3* | CARTIRTVRVGDGFDLW | 60  (0.077%) | 67  (0.007%) | 41644  (5.588%) | 5490  (0.653%) |
| 2 | *IGHV6-1* | *IGHJ6* | CARGGIAVSNGRGPRVMDVW | 10  (0.013%) | 534  (0.058%) | 10203  (1.369%) | 741  (0.088%) |
| 3 | *IGHV5-51* | *IGHJ6* | CARLSGSAIAPGRAYNYFGMDVW | 11  (0.014%) | 15  (0.002%) | 7021  (0.942%) | / |
| 4 | *IGHV5-51* | *IGHJ6* | CARISGSALPPGRAYYYYGMDVW | 13  (0.017%) | 4  (0.0004%) | 4776  (0.641%) | / |
| 5 | *IGHV4-39* | *IGHJ4* | CARHRGIAVVSDYW | 20  (0.026%) | 217  (0.023%) | 4528  (0.608%) | 1  (0.0001%) |
| 6 | *IGHV4-39* | *IGHJ6* | CARRGSPYYHYLDVW | 4  (0.005%) | 7  (0.001%) | 4072  (0.546%) | / |
| 7 | *IGHV4-4* | *IGHJ4* | CARGVALISGKRDLNFDYW | 4  (0.005%) | 2  (0.0002%) | 3810  (0.511%) | / |
| 8 | *IGHV4-39* | *IGHJ5* | CARHLAGHIAVAGAEFW | 26  (0.033%) | 10  (0.001%) | 3332  (0.447%) | / |
| 9 | *IGHV6-1* | *IGHJ4* | CARQYASVLGYW | 5  (0.006%) | 13  (0.001%) | 2892  (0.388%) | / |
| 10 | *IGHV4-4* | *IGHJ4* | CARGHGGEDAYTVVPTDW | 5  (0.006%) | 2  (0.0002%) | 2592  (0.348%) | / |
| 11 | *IGHV4-59* | *IGHJ4* | CARERPGTSSFEYW | / | 4777  (0.516%) | 1296  (0.174%) | 3  (0.0004%) |
| 12 | *IGHV4-59* | *IGHJ6* | CAREMSNSSGPAILYYYYMDVW | / | 1205  (0.130%) | 1184  (0.159%) | 63  (0.007%) |

**Supplementary table 11 Characterization of specific clusters of sample L3 of IgG-H CDR3 repertories at T1, T2, T3 and T4**

| ID | *IGHV* | *IGHJ* | CDR3 sequence | T1 | T2 | T3 | T4 |
| --- | --- | --- | --- | --- | --- | --- | --- |
| 1 | *IGHV1-18* | *IGHJ6* | CARAHGFYHYMDVW | 11  (0.051%) | 757  (0.266%) | 8046  (4.118%) | 13  (0.004%) |
| 2 | *IGHV4-39* | *IGHJ4* | CATQGPTTDTTGYYPIDYW | 10  (0.046%) | 4  (0.001%) | 5888  (3.014%) | / |
| 3 | *IGHV6-1* | *IGHJ4* | CARDRQEAPRDFDSW | 38  (0.176%) | 404  (0.142%) | 2958  (1.514%) | / |
| 4 | *IGHV6-1* | *IGHJ5* | CARGYGSGSYDCW | 6  (0.028%) | 364  (0.128%) | 2834  (1.451%) | / |
| 5 | *IGHV4-55* | *IGHJ4* | CGRLRGYFDFW | 2  (0.009%) | 171  (0.060%) | 2556  (1.308%) | / |
| 6 | *IGHV1-18* | *IGHJ5* | CARDWAYITSQDCFDPW | 10  (0.046%) | 85  (0.030%) | 2268  (1.161%) | / |
| 7 | *IGHV4-4* | *IGHJ4* | CAIPIHIRGYTNW | 2  (0.009%) | 110  (0.039%) | 2242  (1.148%) | / |
| 8 | *IGHV5-51* | *IGHJ4* | CARQGDSRRDYW | 7  (0.033%) | 6  (0.002%) | 2091  (1.070%) | / |
| 9 | *IGHV6-1* | *IGHJ4* | CARGWNNFDYW | 2  (0.009%) | 4  (0.001%) | 1757  (0.899%) | / |
| 10 | *IGHV4-39* | *IGHJ4* | CARRGREEYDQHFDHW | 55  (0.255%) | 4  (0.001%) | 1738  (0.890%) | / |
| 11 | *IGHV2-26* | *IGHJ4* | CARKGRGYSYGPFDSW | / | 222  (0.078%) | 249  (0.127%) | / |
| 12 | *IGHV5-51* | *IGHJ4* | CASGLQYESW | / | 166  (0.058%) | 790  (0.404%) | / |
| 13 | *IGHV4-4* | *IGHJ5* | CSRSLPSHASGWFESW | / | 163  (0.057%) | 908  (0.465%) | 8  (0.003%) |
| 14 | *IGHV4-59* | *IGHJ4* | CARGRWGDVSGGHIPFDHW | / | 148  (0.052%) | 1168  (0.598%) | / |

**Supplementary table 12 Characterization of specific clusters of sample L4 of IgG-H CDR3 repertories at T1, T2, T3 and T4**

| ID | *IGHV* | *IGHJ* | CDR3 sequence | T1 | T2 | T3 | T4 |
| --- | --- | --- | --- | --- | --- | --- | --- |
| 1 | *IGHV5-51* | *IGHJ4* | CAKSGAAGDYSDAW | 52  (0.020%) | 3361  (0.763%) | 35417  (4.201%) | / |
| 2 | *IGHV4-4* | *IGHJ6* | CARTLTGDHVGYYGVDVW | 59  (0.023%) | 8  (0.002%) | 31819  (3.774%) | / |
| 3 | *IGHV4-39* | *IGHJ3* | CASGRDTYDAFHVW | 2  (0.001%) | 34  (0.008%) | 26202  (3.108%) | / |
| 4 | *IGHV4-4* | *IGHJ4* | CARGWGGYYTDW | 9  (0.004%) | 2  (0.0005%) | 8664  (1.028%) | / |
| 5 | *IGHV4-59* | *IGHJ6* | CARTLTGDEVGYYGVDVW | 4  (0.002%) | 43  (0.010%) | 7198  (0.854%) | / |
| 6 | *IGHV4-39* | *IGHJ5* | CARHADVYSWFDPW | 18  (0.007%) | 2  (0.0005%) | 6059  (0.719%) | / |
| 7 | *IGHV4-39* | *IGHJ3* | CARQADIYDAFDIW | 10  (0.004%) | 19  (0.004%) | 5949  (0.706%) | / |
| 8 | *IGHV5-51* | *IGHJ5* | CARHSGGVRNWFDSW | 3  (0.001%) | 48  (0.011%) | 5045  (0.598%) | 29  (0.003%) |
| 9 | *IGHV4-4* | *IGHJ4* | CASAQDVYNSLDYW | 2  (0.001%) | 4  (0.001%) | 4522  (0.536%) | / |
| 10 | *IGHV4-4* | *IGHJ2* | CASASLMSGTNWDWYFDLW | 25  (0.010%) | 1  (0.0002%) | 4330  (0.514%) | 7  (0.001%) |

**Supplementary table 13 Characterization of specific clusters of sample L5 of IgG-H CDR3 repertories at T1, T2, T3 and T4**

| ID | *IGHV* | *IGHJ* | CDR3 sequence | T1 | T2 | T3 | T4 |
| --- | --- | --- | --- | --- | --- | --- | --- |
| 1 | *IGHV4-4* | *IGHJ4* | CATQATGHRSPFDYW | 52  (0.041%) | 65  (0.008%) | 37028  (2.484%) | / |
| 2 | *IGHV4-4* | *IGHJ5* | CAKDRGYGDRMGFLHW | 2  (0.002%) | 2  (0.0003%) | 18506  (1.241%) | / |
| 3 | *IGHV1-8* | *IGHJ6* | CARGNRPVGPYYFGMDVW | 10  (0.008%) | 27  (0.003%) | 17031  (1.142%) | / |
| 4 | *IGHV4-39* | *IGHJ6* | CGRLFRDQPYGMDVW | 30  (0.024%) | 18  (0.002%) | 14648  (0.982%) | / |
| 5 | *IGHV5-51* | *IGHJ3* | CARRPDPPYSVAVFDIW | 15  (0.012%) | 12  (0.002%) | 10435  (0.700%) | / |
| 6 | *IGHV4-4* | *IGHJ4* | CARLVLTGMIPARGYFDLW | 9  (0.007%) | 10  (0.001%) | 1005  6(0.674%) | / |
| 7 | *IGHV4-4* | *IGHJ4* | CARGRNWNYESHFDYW | 2  (0.002%) | 6  (0.001%) | 9773  (0.655%) | / |
| 8 | *IGHV5-51* | *IGHJ4* | CVTSSRRMFDYW | 30  (0.024%) | 897  (0.114%) | 8562  (0.574%) | / |
| 9 | *IGHV4-4* | *IGHJ4* | CAKDRGYGDRTGFIYW | 18  (0.014%) | 4  (0.001%) | 8559  (0.574%) | / |
| 10 | *IGHV4-4* | *IGHJ3* | CARFDGSGPRRGSFDVW | 24  (0.019%) | 2  (0.0003%) | 7834  (0.525%) | / |
| 11 | *IGHV4-4* | *IGHJ2* | CVRQYALGKWYFDLW | / | 935  (0.119%) | 3200  (0.215%) | / |
| 12 | *IGHV4-39* | *IGHJ4* | CARRPYSGHDEGGYW | / | 900  (0.115%) | 5049  (0.339%) | / |
| 13 | *IGHV1-67* | *IGHJ4* | CAREQDKTCYFDYW | / | 762  (0.097%) | 1156  (0.078%) | / |

**Supplementary table 14 HBV vaccine-specific sequences that have been reported in the literature mapped to this experiment**

| ID | CDR3 AA sequence | ID | CDR3 AA sequence |
| --- | --- | --- | --- |
| 1 | CGRSRLNSYGLDVW(13) | 9 | CAREGTSTEAQLGYYYGMDVW(15) |
| 2 | CARRSLQYRYGLDVW(13) | 10 | CARFGDWDGYDYHYGMDVW(15) |
| 3 | CARGMRDAFDTW(15) | 11 | CTRRGYYGSGSYYGDYW(33) |
| 4 | CARQLTKYYDSDIPYQSGLDAFDVW(15) | 12 | CARQRYGGYYGSGSYGPYYSMDAW(15) |
| 5 | CAKERGYCSGLNWRDDAFDFW(13) | 13 | CGRHRGGEVATMGAFDIW(15) |
| 6 | CAKDIASWYYYGMDVW(12) | 14 | CARPLSLVPAAEHNWFDPW(15) |
| 7 | CARRDYGGNPLRPWGYYYGMDVW(15) | 15 | CARDGRLNWFDPW(15) |
| 8 | CAKFGDWDGTDIYYGMDVW(15) |  |  |
